# The displacement of teeth and stress distribution on periodontal ligament under different upper incisors proclination with clear aligner in cases of extraction: a finite element study

**DOI:** 10.1186/s40510-023-00491-2

**Published:** 2023-11-20

**Authors:** Fujia Kang, Yumiao Wu, Yuchen Cui, Jiamin Yuan, Zhiqiang Hu, Xianchun Zhu

**Affiliations:** Department of Orthodontics, Hospital of Stomatology, Jilin University, Jilin University, Changchun, Jilin China

## Abstract

**Objectives:**

To investigate the displacement of dentition and stress distribution on periodontal ligament (PDL) during retraction and intrusion of anterior teeth under different proclination of incisors using clear aligner (CA) in cases involving extraction of the first premolars.

**Methods:**

Models were constructed, consisting of the maxilla, PDLs, CA and maxillary dentition without first premolars. These models were then imported to finite element analysis (FEA) software. The incisor proclination determined the division of the models into three groups: Small torque (ST) with U1-SN = 100°, Middle torque (MT) with U1-SN = 110°, and High torque (HT) with U1-SN = 120°. Following space closure, a 200 g intrusion force was applied at angles of 60°, 70°, 80°, and 90° to the occlusal plane, respectively.

**Results:**

CA therapy caused lingual tipping and extrusion of incisors, mesial tipping and intrusion of canines, and mesial tipping of posterior teeth in each group. As the proclination of incisors increased, the incisors presented more extrusion and minor retraction, and the teeth from the canine to the second molar displayed an increased tendency of intrusion. The peak Von Mises equivalent stress (VMES) value successively decreased from the central incisor to the canine and from the second premolar to the second molar, and the VMES of the second molar was the lowest among the three groups. When the angle between the intrusion force and occlusal plane got larger, the incisors exhibited greater intrusion but minor retraction.

**Conclusions:**

The "roller coaster effect" usually occurred in cases involving premolar extraction with CA, especially in patients with protruded incisors. The force closer to the vertical direction were more effective in achieving incisor intrusion. The stress on PDLs mainly concentrated on the cervix and apex of incisors during the retraction process, indicating a possibility of root resorption.

## Introduction

CAs are transparent appliances produced using three-dimensional (3D) printing technology, utilizing computer-aided design and computer-aided manufacturing based on the 3D data from the oral scanner and CBCT [1]. CA provides many advantages over the fixed appliance, such as comfort, esthetics, and hygiene. Along with the continuous improvement in material performance, the upgrading and optimization of attachment shape and function [2], and the advancements in tooth movement track, the indications of CA are expanding. In cases of mild dentition crowding, dentition space and molar distalization, CA can achieve the clinical effects equivalent to those of fixed appliance [3–7], with a high level of predictability.

The application of orthodontic force by CA is based on the rebound force generated after placing the appliance on the crown. Since CAs are removable appliances, a certain degree of elasticity is necessary. Additionally, personal compliance and the height of the clinical crown varies among individuals. Consequently, CAs have limitations in achieving desired teeth movements, particularly in cases involving premolar extraction, remain insurmountable [8]. The phenomenon known as the "roller coaster effect" occurs during the process of space closure [9, 10]. Ren [11] reported undesired tooth movements compared to the intended tooth movements, including mesial movement (2.2 mm), mesial tipping (5.4°), and intrusion (0.45 mm) of first molars; distal tipping (11.0°), lingual tipping (4.4°), and distal rotation of canines (4.9°); as well as lingual tipping (10.6°) and extrusion (1.5 mm) of incisors. The presence of occlusal interference in the anterior teeth obstructs the retraction of anterior teeth. The space closure process can be restarted after the opening of bite in the anterior teeth area. However, Muro MP [12] demonstrated that intrusion was the least predictable movement with CA therapy. In a meta-analysis by Rossini [13], the efficacy of intrusion movement was reported to be 45% for maxillary central incisors and 33% for lateral incisors. To facilitate intrusion movement with CA, several methods have been employed, including overcorrection, additional anchorage such as mini-implants, and specific attachments such as shape-optimized attachments, bite turbos, and bite ramps. It is important to note that these methods have been validated in clinical research and FEA [14–18].

The arrangement of teeth varies among individuals, and the relationship between the proclination of incisors and the efficiency of movement remain unclear. FEA is an engineering technology that enables the simulation of initial displacement and VMES distribution on the teeth, PDLs and alveolar bone [19, 20]. To investigate the comprehensive tooth movement during retraction and explore the stress distribution on PDL to guide the safe application of CA in clinical practice, a high-precision three-dimensional finite element model was constructed, divided into three groups according to the proclination of incisors. An intrusion force of 200 g, angled at 60°, 70°, 80° and 90° to the occlusal plane was applied on the CA.

## Materials and methods

### FE model establishment

**Fig. 1:**
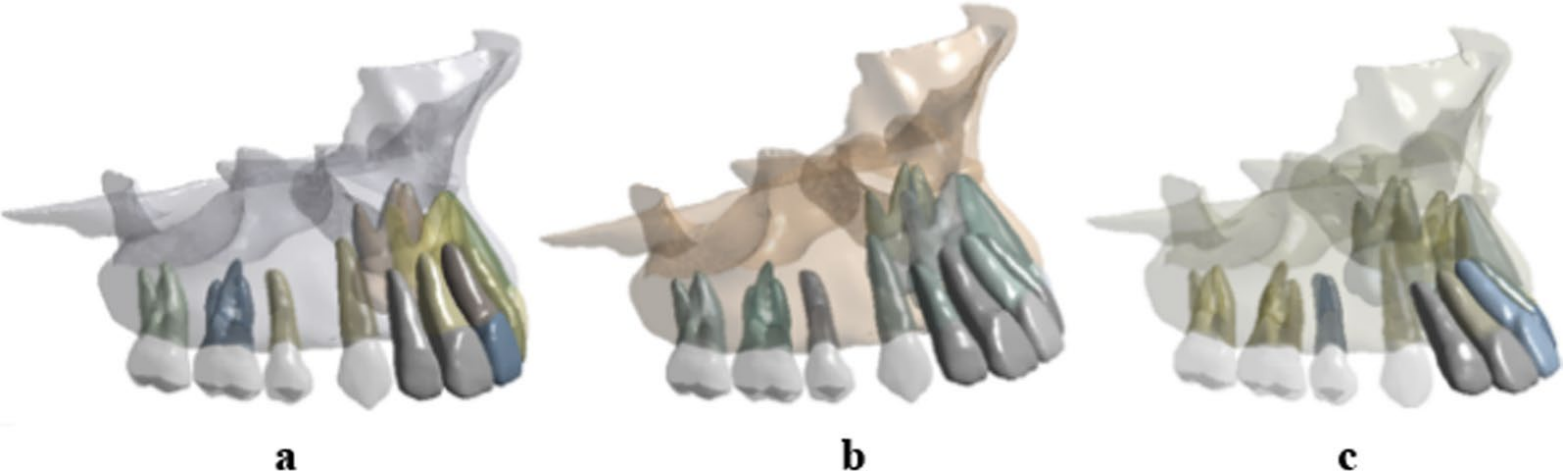
The 3D maxillary models of three different incisors proclination conditions. **a** Small torque(ST):U1-SN = 100. **b** Middle torque(MT): U1-SN = 110. **c** High torque(HT): U1-SN = 120°

This study has received approval from the Ethics Committee of Jilin University Stomatological Hospital (No. 56 in 2022) and informed consent was obtained from the patient. One patient with moderately crowded and permanent dentition was selected as the subject for this study. The size and shape of the teeth were standard, and the extraction of four first premolars was planned for orthodontic treatment. CBCT (Planmeca Pro Max 3D, Finland) of the patient was collected and imported into Mimics software (Mimics Research v21.0, Materialise, Leuven, Provincie Vlaams-Brabant, Belgium). Teeth and maxilla models were segmented using threshold operation and region growth command. The resulting model files were then imported into 3-Matic software (3-Matic Research v13.0, Materialise, Leuven, Provincie Vlaams-Brabant, Belgium). The first premolars on both sides were removed and the remaining teeth were manually aligned with a 0.5 mm gap between the canines and lateral incisors. The central incisors and lateral incisors were arranged according to their proclination: Small torque (ST): U1-SN = 100°, Middle torque (MT): U1-SN = 110°, and High torque (HT): U1-SN = 120°, as shown in Fig. 1. Following the previous study [21, 22], the PDL was set with a 0.2 mm offset from the roots. The tooth movement step was designed to be 0.2 mm, retracting along the occlusal plane. The target dentition was modeled by adducting the incisors along the occlusal plane. The CA model was then created by offsetting the external surface of the tooth crown of the target dentition with a thickness of 0.7 mm. The completed FE model consisted of 26 solid units:12 teeth, 12 PDLs, clear aligner and maxillary bone.

### Model meshing and boundary conditions setting

**Fig. 2:**
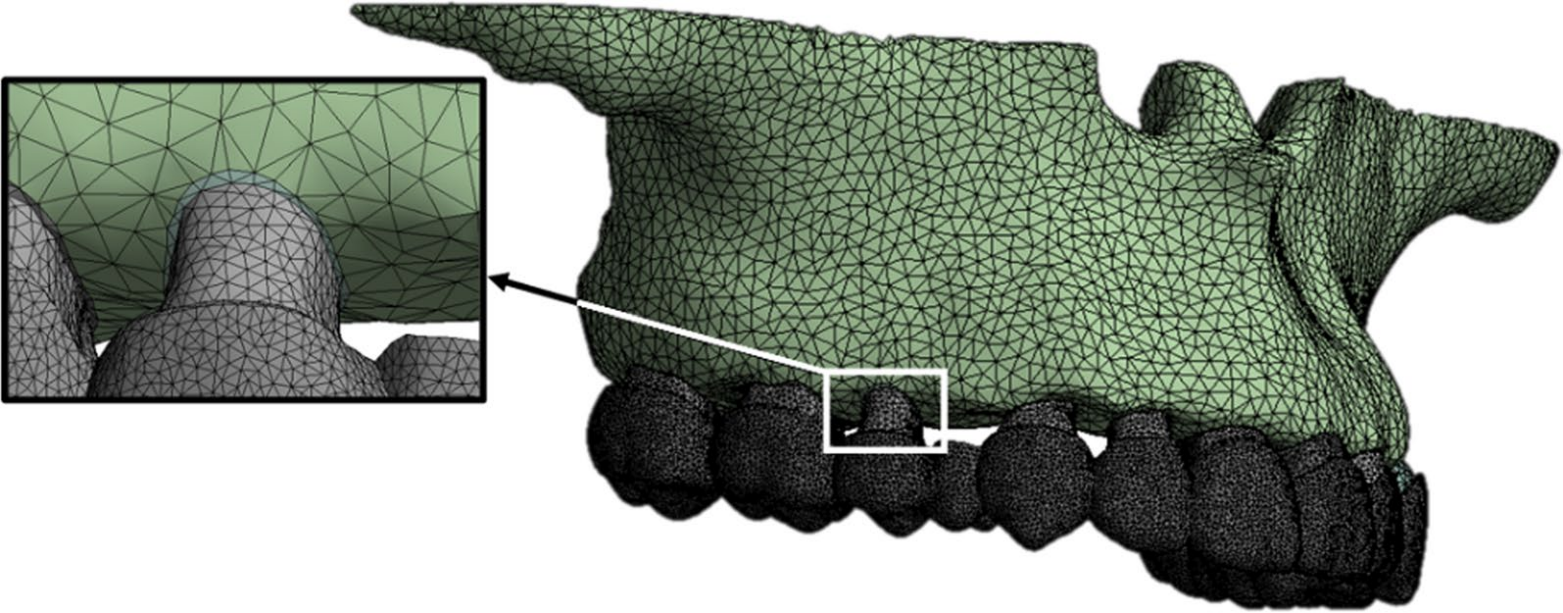
A 3D finite element model in the orthodontic treatment situation, the nodes among teeth, PDLs and alveolar bone was shared

The models were assembled and then meshed into second-order tetrahedron elements, as displayed in Fig. 2, resulting in a total of 426,368 elements and 790,664 nodes. "Bonded" contact was set between the roots and PDLs, as well as between the PDLs and alveolar bone. "Frictional" contact was set between the external surface of the clinical crown and the internal surface of the CA. The friction factor was set as 0.2 based on the reported literature [23]. The upper surface of maxilla was "fixed", restricting all degrees of freedom for the elements. With these settings [24], convergence of the contact calculations for the model was achieved.

### Material parameter assignment and force loading

**Fig. 3:**
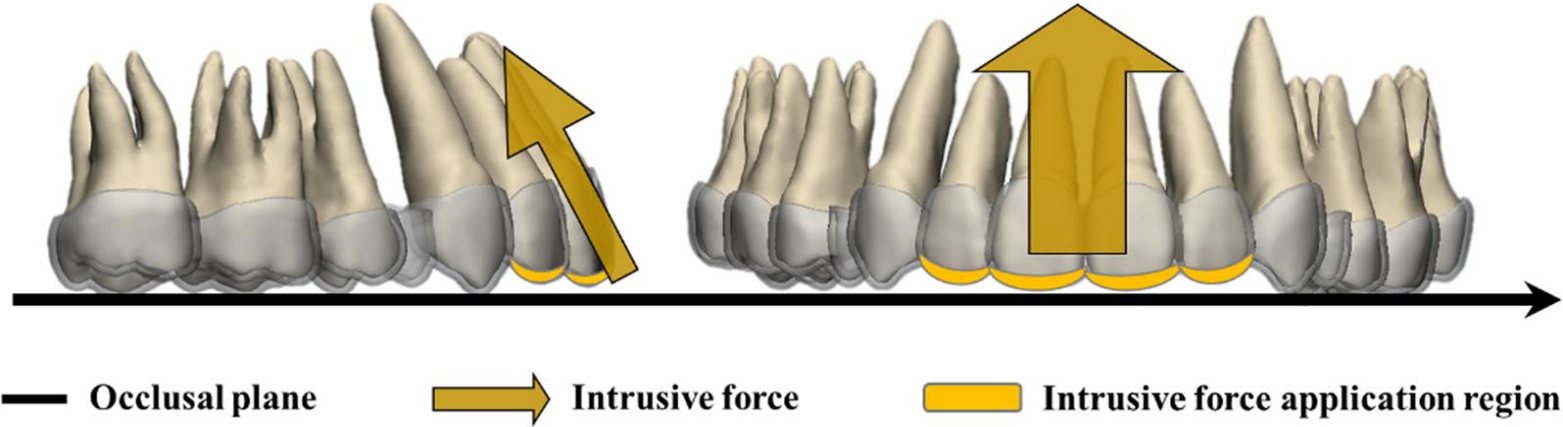
The intrusion force was applied on the CA

The biological materials involved in this study were simplified as linear elastomers; the material parameters were obtained from previous literature, as shown in Table 1 below. To load the orthodontic force, a "two-step method" [23, 25] was employed. This involved applying displacement to the incisors, moving them from the target position to the initial position, extracting the stress results of the CA, and then applying those results to the teeth in initial position. A mini-implant was inserted between the central incisors, approximately 8 mm from the top of the alveolar ridge. An intrusive force of 200 g was applied to the CA using the mini-implant. The angle between the intrusive force and occlusal plane varied at 60°, 70°, 80° and 90° respectively (see Fig. 3). Since the stress condition of the mini-implant was not further discussed, it was excluded from the model.

**Table 1:** Material properties of the FE model

	Young's modulus (MPa)	Poisson's ratio	References
Teeth	19,600	0.3	[15, 26]
Alveolar bone	13,700	0.3	[21, 23]
PDL	0.68	0.45	[14, 24]
CA	528	0.36	[27]

### Establishment of the coordinate system and observation values

**Fig. 4:**
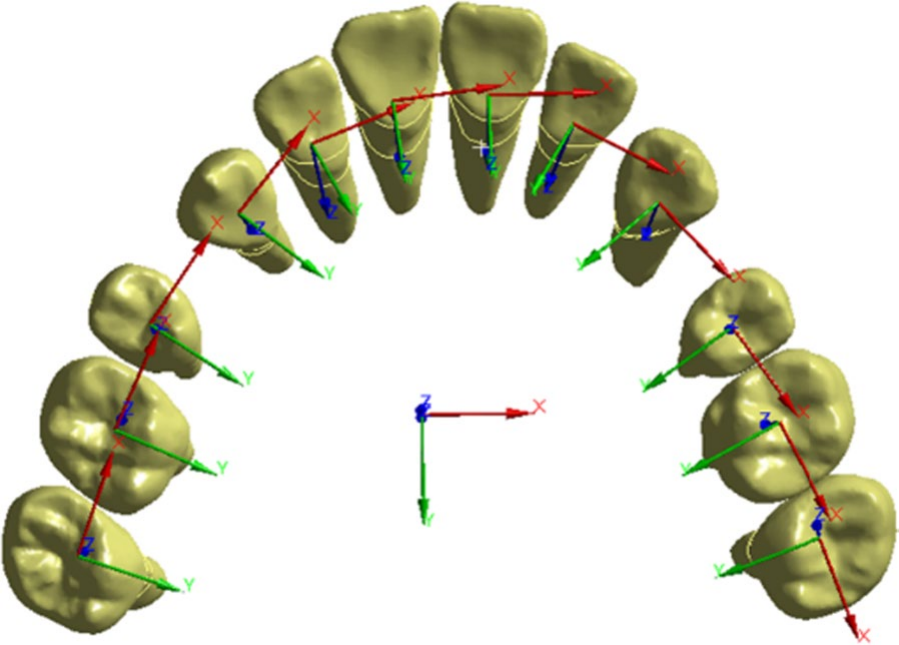
Local coordinate system for each tooth and global coordinate system based on occlusal plane

Local coordinate systems for the teeth were established to define the x, y and z axes (Fig. 4). The origin of the coordinate system was positioned at the center of the tooth, with the long axis of the tooth body defined as the *z*-axis. The positive direction of the *z*-axis indicated the gingival side, the positive direction of the *x*-axis indicated the mesial side, and the positive direction of the *y*-axis indicated the lingual side. Besides, a global coordinate system was defined on the occlusal plane (*x*-*o*-*y* plane), with the *z*-axis pointing upwards and *y*-axis pointing backwards. The analysis focused on determining the total and directional displacement of the teeth and VMES on PDLs. Furthermore, the average displacement of the crown and root were calculated separately to represent their respective overall displacement tendencies.

## Results

**Fig. 5:**
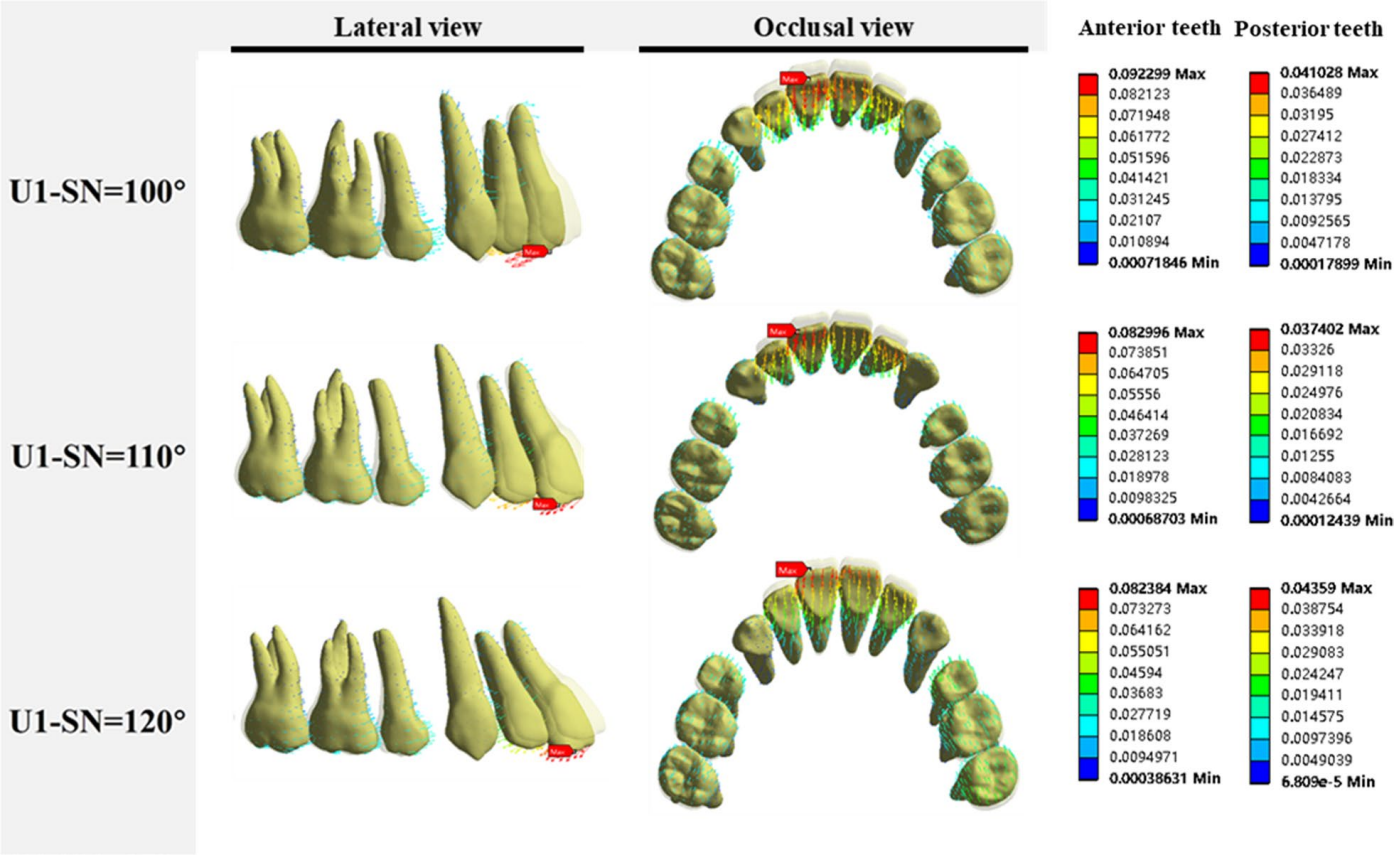
Displacement vectors of dentition under three groups

During the en masse retraction of incisors, the force systems and behaviors of both anterior and posterior teeth were recorded. The results showed a similar pattern of tooth movement among three groups (as shown in Fig. 5). Besides, the movement patterns of the bilateral identical teeth were generally symmetrical. The incisors exhibited lingual tipping and extrusion, while the root apex moved slightly labially compared to the incisor edges. The canines experienced intrusion and lingual rotation on the mesial side, whereas the teeth from the second premolar to the second molar exhibited mesial tipping. In all three groups, the maximum displacement in the dentition occurred at incisal edge of the central incisor. The magnitude of displacement decreased with the increase in the incisors' proclination, measuring 9.92 × 10^−2^ mm, 8.30 × 10^−2^ mm, 8.24 × 10^−2^ mm for the respective groups. However, the posterior teeth did not follow a similar pattern, with maximum displacement values of 4.10 × 10^−2^ mm, 3.74 × 10^−2^ mm and 4.36 × 10^−2^ mm, respectively. Besides, there was only a small amount of movement observed in the canines.

A comprehensive data table (Table 2) was established to quantitatively analyze the movement patterns of the crown and root. From the vertical view, the central incisor exhibited greater extrusion than the lateral incisor, and the extent of extrusion increased from the ST group to the HT group. Similarly, the teeth from canine to the second molar gradually exhibited an increased intrusion tendency from ST group to HT group. In the sagittal view, the central incisor demonstrated greater lingual movement compared to the lateral incisor, with a decreasing trend from the MT group to the HT group. The overall dental arch displayed a narrowing trend, except for the second premolars which exhibited slight buccal movement. The mesial movement of the posterior teeth decreased from the second premolar to the second molar, with no significant difference among the three groups.

**Table 2:** Directional teeth movement under three groups

	17	16	15	14	13	12	11	21	22	23	24	25	26	27
*ST group (U1-SN* = *100°)*														
Extrusion/intrusion (crown)	0.59E	0.19E	0.11E	-	0.80I	1.24E	3.15E	2.91E	1.53E	0.77I	-	0.32E	0.08E	0.08E
Buccal/lingual movement (crown)	0.72L	0.83L	0.09B	-	0.44L	4.90L	6.43L	5.74L	4.73L	0.58L	-	0.49L	0.54L	0.36L
Mesial/distal movement (crown)	1.56 M	1.90 M	2.69 M	-	0.41D	2.45D	0.92D	0.27D	2.51D	0.39D	-	3.00 M	2.22 M	1.83 M
Extrusion/intrusion (root)	0.36E	0.05E	0.27E	-	1.05I	0.65I	0.55E	0.45E	0.32I	1.11I	-	0.37E	0.06E	0.35E
Buccal/lingual movement (root)	0.07L	0.11L	0.11B	-	0.16B	0.39L	0.86L	0.62L	0.35L	0.17B	-	0.02L	0.04L	0.02B
Mesial/Distal movement (root)	0.28 M	0.52 M	0.65 M	-	0.12 M	0.21D	0.35D	0.12D	0.44D	0.00	-	0.92 M	0.54 M	0.33 M
*MT group (U1-SN* = *110°)*														
Extrusion/intrusion (crown)	0.55E	0.01E	0.02I	-	0.74I	2.26E	3.45E	2.84E	2.30E	0.67I	-	0.24I	0.07I	0.60E
Buccal/lingual movement (crown)	0.42L	0.51L	0.17B	-	0.04L	4.34L	5.59L	5.13L	5.13L	0.40L	-	0.60B	0.24L	0.20L
Mesial/distal movement (crown)	1.91 M	1.91 M	2.65 M	-	0.75 M	2.30D	1.18D	1.15D	0.51D	0.50 M	-	2.51 M	2.02 M	1.68 M
Extrusion/intrusion (root)	0.37E	0.09I	0.18E	-	0.80I	0.08I	0.39E	0.04E	0.12I	0.64I	-	0.05I	0.10I	0.37E
Buccal/lingual movement (root)	0.01B	0.08L	0.05L	-	0.18B	0.40L	0.58L	0.73L	0.42L	0.01B	-	0.10B	0.01B	0.03B
Mesial/Distal movement (root)	0.29 M	0.45 M	0.57 M	-	0.09 M	0.27D	0.06 M	0.18D	0.31 M	0.07 M	-	0.52 M	0.43 M	0.32 M
*HT group (U1-SN* = *120°)*														
Extrusion/intrusion (crown)	0.44E	0.11I	0.12I	-	0.18I	2.32E	3.94E	4.04E	2.43E	0.47I	-	0.12I	0.08I	0.48E
Buccal/lingual movement (crown)	0.17L	0.26L	0.14B	-	0.65L	3.69L	5.74L	5.37L	4.07L	0.56 L	-	1.13B	0.22L	0.56L
Mesial/distal movement (crown)	1.65 M	1.97 M	2.55 M	-	0.37 M	1.66D	0.91D	0.19 M	1.77D	0.10 M	-	2.96 M	2.53 M	2.40 M
Extrusion/intrusion (root)	0.31E	0.11I	0.09I	-	0.29I	0.32I	0.19E	0.37E	0.31I	0.68I	-	0.09I	0.05E	0.20E
Buccal/lingual movement (root)	0.03B	0.05L	0.11L	-	0.20L	0.08L	0.49L	0.38L	0.24L	0.06L	-	0.22B	0.05L	0.44 L
Mesial/Distal movement (root)	0.26 M	0.36 M	0.48 M	-	0.22D	0.09D	0.10D	0.08 M	0.12D	0.08D	-	0.65 M	0.40 M	0.40 M

The displacement above is measured at 10^−2^ mm. Crown movement is the average displacement of clinical crown; similarly, the root movement is the average displacement of clinical root

**Fig. 6:**
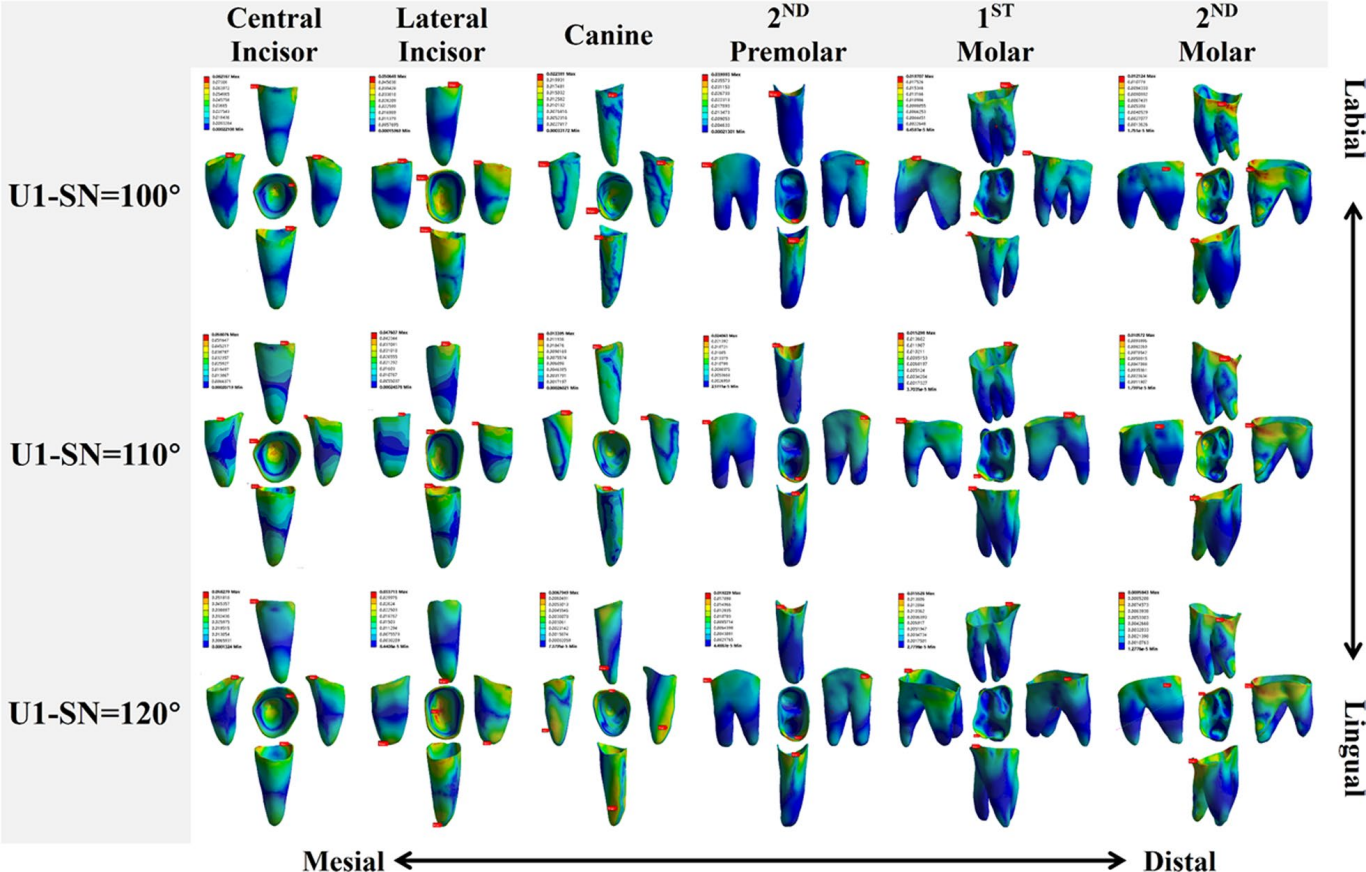
The stress distributions of PDLs of maxillary teeth

The distribution’s pattern of VMES on the PDLs of the right maxillary teeth among three groups was observed, as shown in Fig. 6. The red areas primarily indicated high VMES values, located at the cervix and apex. Conversely, the blue areas represented low VMES values. In each group, it was observed that the peak stress value decreased sequentially from the central incisor to the canine, and from the second premolar to the second molar. Interestingly, the peak stress value of the canine was typically smaller than that of the second premolar. In the anterior tooth region, apart from the central incisor, there was a decreasing trend in peak stress from the MT group to the HT group.

**Fig. 7:**
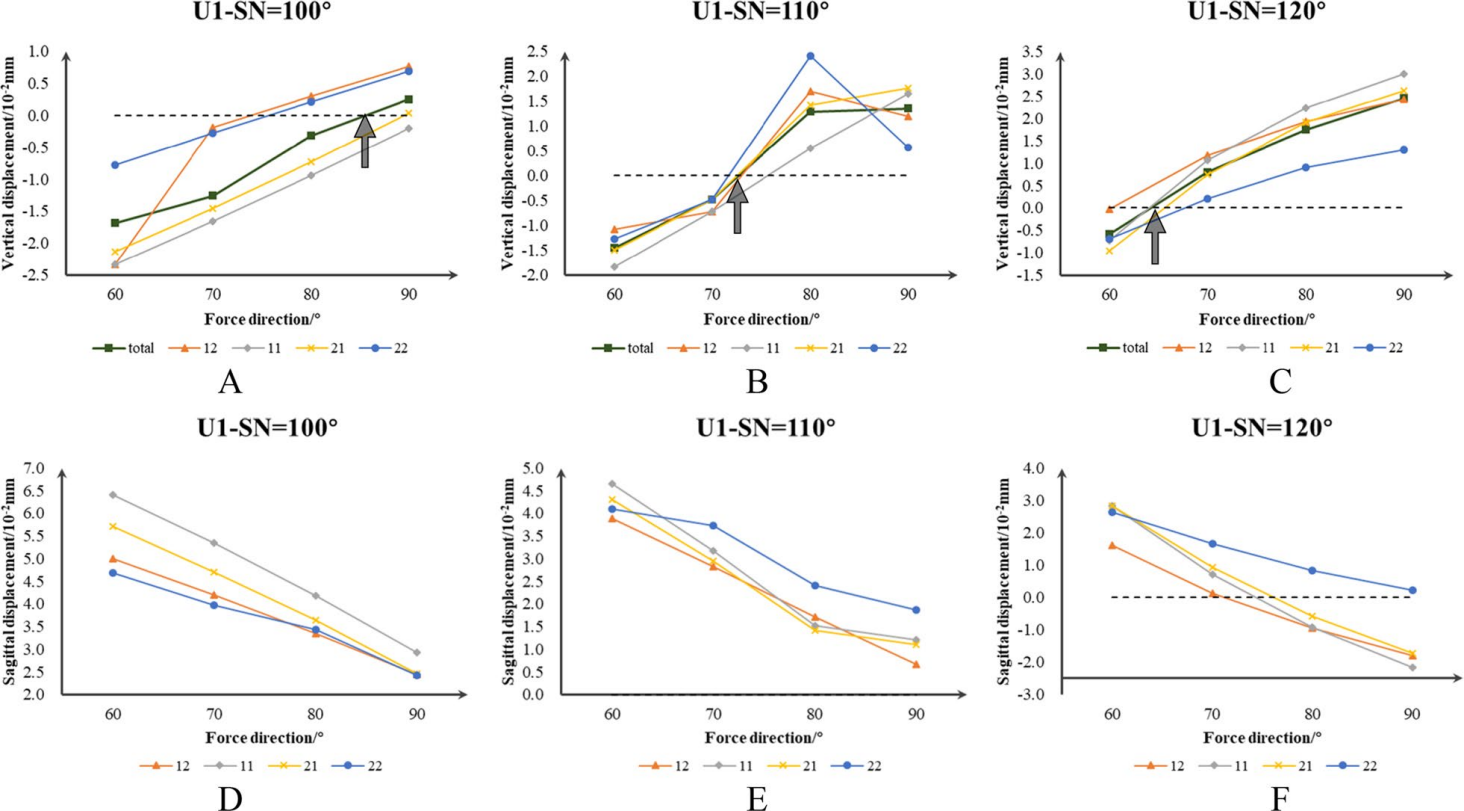
Vertical and sagittal displacement of incisors in three groups under intrusion force. **A**–**C** Vertical displacement of incisors under ST, MT and HT group, the "average" means the average displacement of total incisors, the gray arrow indicates that the total amount of displacement amounted to zero. **D**–**F** Sagittal displacement of incisors under ST, MT and HT group

The line graph in Fig. 7 illustrated the variations in vertical and sagittal displacement caused by different angles of intrusion force among various groups. The *x*-axis represented the angle of intrusion force, while the *y*-axis indicated the displacement of the incisors. The line graph depicting vertical displacement exhibited a gradual upward trend as the angle of the intrusion force increased. Conversely, the line graph representing sagittal displacement displayed a gradual downward trend as the angle of intrusion force increased. Regarding the average displacement of incisors, an intrusion force ranging from 80° to 90° could control the vertical position of incisors in ST group, while the intrusion force of 70°–80° and 60°–70° was respectively effective in MT group and HT group. Additionally, an intrusion force of 60° enabled the maximum adduction movement in all groups.

## Discussion

To figure out the "roller coaster effect", two basic types of tooth movement must be distinguished: bodily movement and rotation. Bodily movement occurs when a force passes through the center of resistance, while rotation occurs when a force does not pass through the center of resistance, resulting in a moment. The magnitude of the moment is equal to the force's magnitude multiplied by the distance from the center of resistance to the force line. For extraction cases, when the incisor is subjected to a single adduction force, it exhibited uncontrolled tipping movement, which leaded to the deepening overbite [28]. To counteract with the adverse incisor movement, torque control and intrusion is necessary.

The FEA model in our study had the following characteristics: (1) the quality of mesh division was excellent and there was no obvious mesh distortion in the analysis results, the second-order tetrahedral element grid was used which was suitable for the biological structure with irregular shape [29]. (2) The model had high biofidelity as it was modeled using high-precision CBCT to restore the actual structure. Additionally, the mesh connection between the teeth, PDLs, and alveolar bone was done in a common node manner [30]. (3) The force application of CA was simulated, where the orthodontic force on the teeth was generated from the elastic deformation of the appliance. (4) The average displacement of crown and root was calculated. FEA provided displacement values of model’s finite elements and nodes, which was more detailed and comprehensive than the previous observation method of selecting certain point [31]. It should be noted that the average displacement of crown was less than that of cusp or incisal edge, whereas the average displacement of the root was larger than that of root apex.

**Fig. 8:**
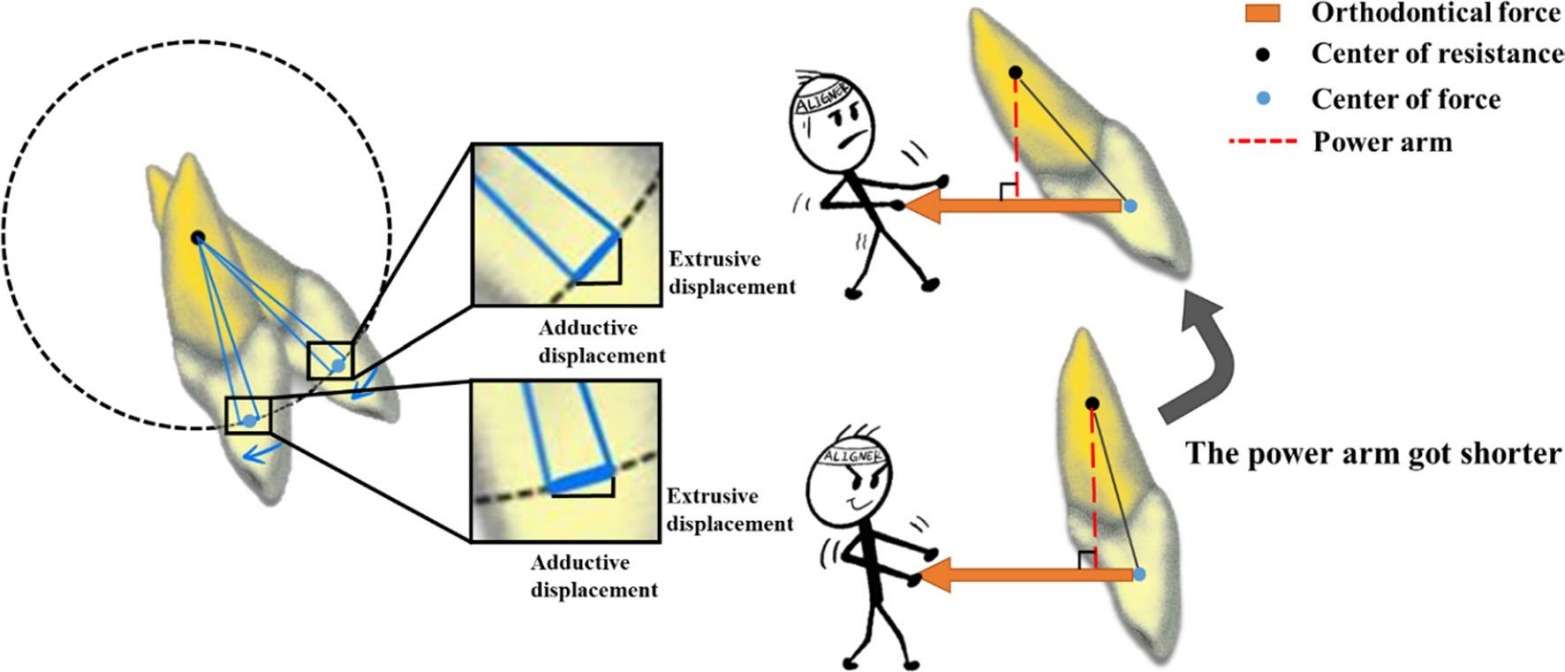
Biomechanical effects of retracting incisors for different proclination of incisors

We observed that as the proclination of incisors increased, the efficiency of adduction movement along the occlusal plane decreased, while the extrusion effect on incisors became more significant. To clarify this issue, we simplified this kind of movement to a circle. The rotation center of incisor constituted the center of the circle, and the movement trajectory of the equivalent CA force application center constituted the arc of the circle (the mechanism above was depicted in Fig. 8). The reduction in length for each CA was same, resulting in the same arc length traversed by the force application center. When the proclination of the incisor increased, the extrusive displacement increased, while the retractive displacement decreased. Another possible reason for decreased retraction was the leverage effect. Because the resistance center, rotation center, and force application center of the incisor remained relatively constant, the incisor could be viewed as a lever with the rotation center as the fulcrum. As the inclination of the anterior teeth increased, the length of the power arm decreased, making the incisor a laborious lever and reducing the efficiency of adduction movement. As a result, patients with protruded incisors, who will experience larger clockwise torque change, are more susceptible to the deep overbite.

**Fig. 9:**
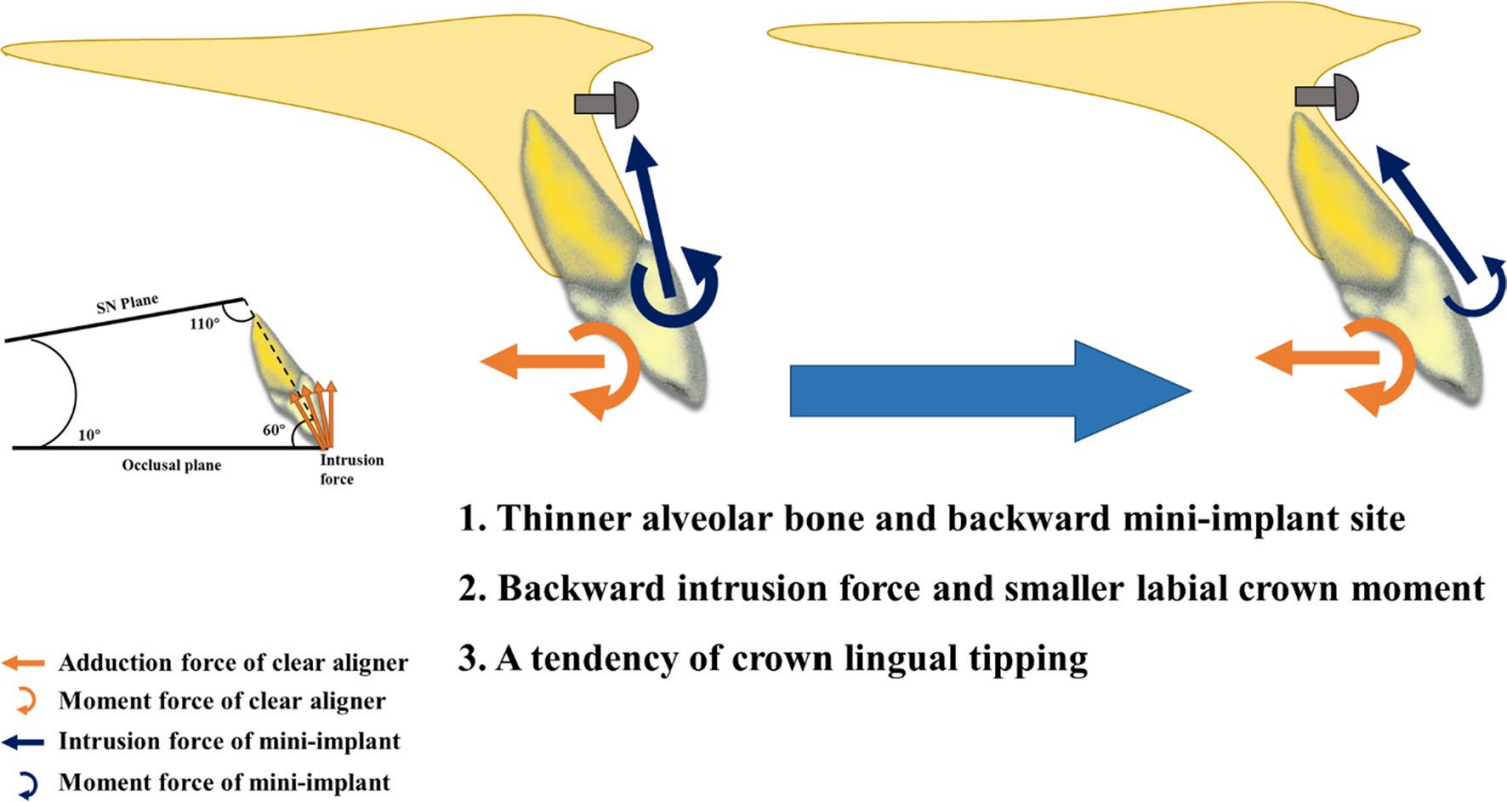
Biomechanical effects of incisors intrusion under different direction of intrusion force

For premolar extraction cases, mini-implants in the anterior alveolar bone guarantee the intrusion. In clinical practice, there are mainly two methods of wearing elastic bands. One involves wearing the elastic band from the mini-implant to the labial surface of the incisors or CA, while the other involves wearing it from the mini-implant to the lingual surface of the incisors or CA. For the later situation, the incisal edge of incisors is subject to a resultant force close to the direction of long axis of incisors. Liu [14] FEA study validated linguo-incisal elastics was more advantageous over labial elastics in achieving incisor bodily intrusion. Our study found that when the angle between the intrusion force and occlusal plane got larger, the intrusion effect got manifest, which is illustrated in Fig. 9. The retraction force from CA caused a lingual crown moment, which giving rising to the extrusion of incisors. For patients with thin alveolar bone, the site for mini-implant site had to be positioned backward. When the angle between the intrusive force and the occlusal plane decreased, the labial crown moment produced by the intrusive force, which could counteract the opposite moment from the CA, also decreased. The appropriate direction for intrusion, which maintained the vertical position of incisors, was consistently 10° labial to the long axis of incisors. Likewise, another FEA analysis [32] revealed that as the IMPA increased from 90° to 110°, the moment of mandibular central incisors changed from a lingual crown moment to a labial crown moment. Additionally, an intrusion force of approximately 50–100 g per side have been proved effective and safe [33, 34]. However, poor control over the inclination of the canine axis with CA results in distal inclination and the formation of a fulcrum in the canine area. This causes occlusal displacement of the CA in the anterior teeth area. Similar effects occur when using the bite-ramp. This occlusal displacement weakens the effect of the intrusion force applied to the anterior teeth. Consequently, a relatively larger intrusion force guarantees the fitting of the CA. Caution should be paid when applying orthodontic force as exceeding the appropriate force level does not increase the efficiency of tooth movement but can result in root absorption and even pulp necrosis. Patients with short roots, poor periodontal conditions, thin gingiva, and older age should receive slightly lower force application during treatment.

Appropriate torque control is essential for ensuring proper intrusion, which guarantees that the root is located in the cancellous bone. In a FEA conducted by Cheng [16], the power ridge was analyzed, revealing that protruded upper anterior teeth require weak torque control. Besides, the thicker aligners should be designed to facilitate bodily retraction of anterior teeth and minimize root resorption [35]. Liu [15] discovered that attachments on canines could enhance root torque control, although their efficiency was less effective for lateral incisors compared to central incisors.

Orthodontic tooth movement is a consecutive process including applying orthodontics force, alveolar bone remodeling and tooth displacement. The FEA analysis method provides a transient effect under certain circumstance, resulting in a displacement tendency smaller than the actual biological displacement [20, 36]. For the evidence at higher level, further clinical trials need to be conducted. What approaches can be employed to achieve bodily intrusion of incisors? How to control the risk of root resorption? Is it feasible to utilize a combination of bite-ramp and intrusion overcorrection as a substitute for the mini-implant? These issues need to be further explored.

## Conclusions

The "roller coaster effect" happened during the process of retracting anterior teeth using CA in the cases involving the extraction of first premolars. When the proclination of incisors increased, the incisors presented more extrusion and minor retraction, and the teeth from canine to the second molar displayed an increased tendency of intrusion. The intrusion force from the mini-implant was proved effective to achieve intrusion. Moreover, when the angle between the intrusion force and occlusal plane got larger, the relative intrusion for incisors was more significant but the retraction movement was hindered. To maintain the vertical position of the incisors, it was recommended to apply a 200 g intrusion force inclined 10° labially relative to the long axis of the incisors. The stress on PDLs mainly concentrated on the cervix and apex during the retraction process, indicating a possibility of root resorption and alveolar defects.

## Data Availability

The data that support the findings of this study are available from the corresponding author and the first author upon reasonable request.

## Abbreviations

PDL: Periodontal ligament
CA: Clear aligner
FEA: Finite element analysis
ST: Small torque
MT: Middle torque
HT: High torque
3D: Three dimensional
VMES: Von Mises equivalent stress
CBCT: Cone-beam computed tomography
